# Dynamic Compressive Mechanical Properties of Polyvinyl Alcohol Fiber-Reinforced Geopolymer Composites

**DOI:** 10.3390/ma19061128

**Published:** 2026-03-13

**Authors:** Mingyang Li, Qi Liu, Yizhong Tan, Fanfei Dai, Shenghui Wang

**Affiliations:** Training Base, Army Engineering University of PLA, Xuzhou 221116, China; lmyaeu@163.com (M.L.); lrk5113@163.com (Q.L.); fanfeidai1017@163.com (F.D.); wangshenghui1226@163.com (S.W.)

**Keywords:** fiber-reinforced geopolymer composites, dynamic compressive strength, split Hopkinson pressure bar, polyvinyl alcohol fiber

## Abstract

Polyvinyl alcohol (PVA) fibers are commonly added to fiber-reinforced geopolymer composites (FRGC) to enhance their properties; however, systematic research on the dynamic mechanical properties of polyvinyl alcohol fiber-reinforced geopolymer composites (PVA-FRGC) is still required. In this study, an orthogonal experimental design was adopted to investigate the effects of the fly ash/slag ratio, fiber length, and fiber volume content on the dynamic mechanical properties (dynamic compressive strength, fragmentation degree, and energy absorption capacity) of PVA-FRGC. A split Hopkinson pressure bar (SHPB) was used to test the dynamic mechanical properties of the material. The results indicate that the fly ash/slag ratio, fiber length, and fiber volume content all exert significant effects on the dynamic compressive strength and energy absorption capacity of PVA-FRGC. The addition of PVA fibers significantly improves the dynamic compressive strength of PVA-FRGC, which reaches 157.52 MPa, 183.26 MPa, and 210.68 MPa under three different strain rates ranging from 75.4 s^−1^ to 179.6 s^−1^, respectively. Although the energy absorption capacity of PVA-FRGC is not significantly improved, the integrity of the specimens after fragmentation is remarkably enhanced. Specifically, under the three load levels, the average particle sizes of PVA-FRGC are 241.43%, 245.04%, and 127.80% higher than those of plain geopolymers, respectively. Considering the comprehensive dynamic mechanical properties, a fly ash/slag ratio of 5:5, a fiber length of 9 mm, and fiber volume content of 2.0% can be regarded as the local optimal mix proportion.

## 1. Introduction

With the transformation of China’s development model, green development has emerged as a central focus of social progress. The raw materials used in the production of traditional concrete impose considerable environmental burdens. In terms of mechanical performance, conventional concrete exhibits compressive strength that is substantially higher than its tensile strength, and it typically fails in a brittle manner [1,2,3,4,5], with limited post-peak deformability. In engineering structures, this mechanical behavior poses significant safety risks, particularly when the structure is subjected to dynamic impact loads, potentially leading to personal injury and property damage. Therefore, it is essential to develop more environmentally friendly materials capable of undergoing large deformations while retaining adequate strength under loading, especially under dynamic impacts.

Fiber-reinforced geopolymer composites (FRGC) represent a novel class of additive manufacturing materials that can serve as alternatives to reinforced concrete in engineering applications [6,7,8,9,10]. These composites offer high strength, ultra-high toughness, and environmental sustainability, showing great promise for use in prefabricated construction, 3D printing, hydraulic engineering, and underground structures. The primary raw material for FRGC is a geopolymer. The mechanism underlying the capacity of fiber-reinforced composites—such as engineered cementitious composites (ECC) and FRGC—to undergo large deformations, exhibit saturated multiple cracking, and demonstrate strain hardening under static loading is rooted in their microscale and mesoscale mechanical properties, governed by both strength and energy criteria [11,12,13,14]. The concept of geopolymers was first proposed in the 1980s. These materials are inorganic polymers characterized by a three-dimensional network structure composed of tetrahedral units of AlO_4_ and SiO_4_. Common precursors for geopolymer synthesis include metakaolin, fly ash, and slag. Among these, fly ash and slag—both industrial byproducts—are widely available and cost-effective, making them commonly used in various geopolymer formulations. Alkali-activated fly ash-based geopolymers have been the most extensively studied [15,16,17,18,19]. For instance, Liu et al. [20] developed a fly ash/slag-based geopolymer using fly ash, slag, and sodium silicate, with sodium hydroxide as an activator. By adjusting the fly-ash-to-slag ratio and the modulus of the alkali activator, they achieved compressive strength of 39.0 MPa and 76.6 MPa at 1 day and 28 days, respectively. Cheng Guodong et al. [21,22] prepared an alkali-activated geopolymer mortar by varying the fly-ash-to-slag ratio, water-to-cement ratio, and water glass modulus. Their results indicated that a fly-ash-to-slag ratio of 3:7, a water glass modulus of 1.6, and a water-to-cement ratio of 0.35 yielded the highest flexural and compressive strength at 28 days—7.8 MPa and 57.2 MPa, respectively. To mitigate the inherent brittleness of geopolymer concrete and broaden its range of applications, Motohiro Ohno et al. [23,24] incorporated polyvinyl alcohol (PVA) fibers into the matrix and conducted cubic compression and dog-bone tensile tests. The results confirmed the material’s ultra-high tensile strain capacity and multiple-cracking behavior. Yi Wang et al. [25] investigated the effects of the curing conditions and fiber content on the properties of PVA-FRGC. In summary, the static mechanical properties of materials are influenced by multiple factors, and current research on the static behavior of PVA-FRGC is relatively comprehensive. However, in underground engineering, support materials are often subjected to impact loads due to disturbances from blasting and rockburst disasters. Owing to its inherent brittleness, ordinary concrete exhibits limited energy absorption capacity under impact loading. When fiber-modified materials are incorporated to improve the toughness, fiber-reinforced concrete demonstrates increased dynamic compressive strength with rising strain rates under impact loading [26,27]. Musaad Zaheer Nazir Khan et al. [28] studied the dynamic mechanical properties of fiber-reinforced slag/fly ash-based geopolymer composites and compared them with unreinforced matrices. Their findings revealed that fiber addition had no significant effect on quasi-static compressive strength; however, under dynamic impacts, the dynamic increase factor (DIF)—defined as the ratio of dynamic to quasi-static compressive strength—was higher for fiber-reinforced composites, and the threshold for the DIF was also elevated. Sami Doner et al. [29] investigated the dynamic mechanical behavior of cement mortar reinforced with waste iron powder. Under high strain rates, the dynamic compressive strength of specimens increased markedly with higher iron powder content, likely due to the toughening effect of the iron particles. Nevertheless, iron powder addition adversely affected mixture fluidity, limiting the maximum content to 20%. Musaad Zaheer Nazir Khan et al. [30] further examined the dynamic compressive performance of a PVA fiber-reinforced geopolymer mortar using split Hopkinson pressure bar (SHPB) tests. The results indicated significant strain rate sensitivity under impact loading, and fiber incorporation substantially improved the material’s fracture behavior. Su J.H. [31] conducted SHPB dynamic compression tests on geopolymer concrete (GPC) and found that steel fibers more effectively enhanced the static compressive strength (by 23.2% compared to plain GPC). All specimens exhibited a positive correlation between strength and the strain rate. Ling Y. [32] performed dynamic compression tests on engineered geopolymer composites (EGC) with varying polyethylene (PE) fiber volume fractions (0% to 2.0%) using SHPB tests. The results showed that EGC with 1.5% fiber content exhibited optimal static and dynamic mechanical properties. Increasing the fiber content significantly enhanced the dynamic compressive strength, and all dynamic parameters were sensitive to the strain rate, increasing with it.

Extensive research has been conducted on PVA fiber-reinforced geopolymers, primarily focusing on the effects of factors such as the fiber volume content, alkali activator, and fly ash/slag ratio on the static mechanical properties of materials, including flexural toughness [33,34,35], tensile strength [36], and fracture toughness [37]. However, among the existing relevant studies [34], relatively few have examined the dynamic impact mechanical properties. Moreover, most of them focus on the influence of a single factor on material properties [38], with scarce research on the coupling effects of multiple factors. Therefore, in this study, a three-factor and three-level orthogonal test was adopted to investigate the effects of the fly ash/slag ratio, fiber length, and fiber volume content on the dynamic compressive strength, damage degree, and energy absorption capacity of PVA fiber-reinforced geopolymer composites under dynamic impact loading. The potential optimal material mix proportion is proposed, and the relative importance of each factor is discussed.

## 2. Materials and Methods

### 2.1. Material and Specimen Preparation

This paper describes a three-factor, three-level orthogonal experiment. By controlling three variables—the fly-ash-to-slag ratio, fiber length, and fiber volume content—this study investigated the dynamic compressive strength and energy absorption capacity of PVA-FRGC under different strain rate conditions using a Φ50 mm split Hopkinson bar (SHPB) test device.

The PVA fiber-reinforced geopolymer composite (PVA-FRGC) employed in this study consisted of fly ash, slag powder, anhydrous sodium silicate, and PVA fibers. The fly ash used in this study was Class F Grade 1 fly ash obtained from Henan Lanke Co., Ltd., Zhengzhou, China. It features a low cost and high reactivity, making it suitable for use as the primary low-calcium Si–Al matrix for specimen preparation. The slag used was S140-grade slag powder produced by Shandong Kangjing New Material Technology Co., Ltd., Jinan, China. Anhydrous sodium silicate powder was selected as the alkali activator. It had a modulus of 1.0, with the content of SiO_2_ being 49.5% and the content of Na_2_O being 50.5%. The PVA fiber was supplied by the Baohualin (BHL) Industrial Development Company, Yongan, China, with a diameter of 39 μm, a density of 1.3 g/cm^3^, tensile strength of 1509 MPa, and an elastic modulus of 37 GPa.

The required mass of each raw material in each group was calculated according to the mix proportion. After weighing, the materials (fly ash and slag) were dry-mixed rapidly in a mixer for 2 min, and the anhydrous sodium silicate powder was pre-dissolved in the weighed water in advance. After uniform mixing, the pre-weighed tap water was added to the mixture, and mixing was continued for 3 min. During this period, the pre-weighed PVA fibers were slowly dispersed into the mixture, and mixing was continued for 5 min [39]. The freshly mixed fiber–geopolymer composite was cast into molds with a size of Φ50 mm × 50 mm and 50 mm × 50 mm × 50 mm, respectively. The specimens were vibrated on a vibrating table for 2 min to ensure sufficient compaction. Its surface was slightly higher than the mold so that there was no loss of size after sanding. Then, the specimens were covered with a plastic film to prevent moisture loss and placed in a standard curing room with humidity above 90% and a temperature of 20 ± 1 °C for curing. After 24 h, the molds were demolded and the specimens were numbered. Curing was continued until 28 days of age. The two ends of the specimens were then ground flat, with the dimensional accuracy controlled within 0.02 mm.

Notably, the mixing process should not be prolonged for more than 10 min (2 + 3 + 5 min for each stage), as this may cause fiber agglomeration [39]; furthermore, the vibration time should not be excessive in order to avoid fiber sedimentation at the bottom of the mold, which would impair their uniform distribution within the specimens.

### 2.2. Orthogonal Test Design

For multi-factor and multi-level tests, to maximize test efficiency and economy, reduce the number of tests, accelerate the test process, and quickly determine the optimal combination, an orthogonal test design can be employed for specimen grouping [40]. In the experiment, the results to be evaluated are defined as “indicators”, while the variables that may affect these indicators are referred to as “factors”. The different conditions tested for each factor are designated as “levels”. This study involved three distinct factors, each of which was set with three levels. To improve the test efficiency and economy, the orthogonal test method was adopted to combine the variables in this study. According to the standard three-factor and three-level orthogonal table, a total of 9 groups of mix proportion combinations were designed. Meanwhile, to verify the reinforcing effect of fibers on the material, 3 groups of pure geopolymers mixed with fibers were additionally designed as control groups, as shown in Table 1. Each of the three levels of every factor appeared three times in all 9 groups of specimens, which ensured that each level was tested equally and avoided systematic errors caused by uneven test frequencies. Additionally, each of the 9 different combinations between any two factors appeared once, satisfying the orthogonality condition. In terms of data processing, analysis of variance (ANOVA) was used to evaluate the significance level of each factor, determine the optimal mix proportion combination within the test range, and provide a reference for subsequent research work.

The three distinct factors involved in this study were the fly-ash-to-slag ratio, fiber length, and fiber volume content, which are denoted as Factors A, B, and C, respectively. Specifically, three levels were selected for the fly-ash-to-slag ratio, namely 7:3, 5:5, and 4:6. This parameter selection was based on the parameters reported in previous related studies [21,41,42], and the three ratios (fly ash-dominant, slag-dominant, and equal proportions) were chosen as the research parameters. For the fiber length, 6 mm, 9 mm, and 12 mm were selected; in previous studies, PVA fibers with lengths of 6–12 mm are mostly used in experiments [43,44], and 6 mm, 9 mm, and 12 mm are the most common standard specifications in the market. Regarding the fiber volume content, 1.0%, 1.4%, and 2.0% were selected, which was also based on the parameters adopted in previous related studies [45,46,47]—most reports in existing research have concentrated the fiber content between 1.0% and 2.0%. However, based on an analysis of existing research, the selected fiber content tends to be 1.0%; combined with the actual situation of the preliminary tests in this study, 1.4% was set as the intermediate value for fiber content. The three factors involved in this study and their respective three levels were orthogonally grouped according to the standard three-factor three-level orthogonal table, and the levels of the above-mentioned factors were denoted as Levels 1, 2, and 3, respectively. The anhydrous sodium silicate content in the materials was 8%, the water-to-matrix ratio was 0.35, and the specimen size was Φ50 mm × 50 mm. The detailed mix proportions of the materials are listed in Table 1.

Sensitivity analysis is a method for evaluating the influences of various factors on the test results in orthogonal experiments; it allows one to quantitatively assess the impacts of each factor on the test indicators and quickly and accurately determine the optimal proportions of each component in material ratio tests. Analysis of variance (ANOVA) is adopted to evaluate the sensitivity of various factors to test results; it can be used to test whether there are significant differences between the means of multiple samples. The basic idea of ANOVA is to decompose the total variance of the observed data into systematic variance caused by changes in factor levels (inter-group variance) and unsystematic variance caused by random errors (intra-group variance) and judge the significance of factor effects by comparing the relative magnitudes of the two variances [40].

In terms of calculation principles, ANOVA first involves calculating the total sum of squares of deviations ($SS_{T}$), which is decomposed into the sum of squares of factors ($SS_{A}$) and the sum of squares of errors ($SS_{e}$). Then, the mean squares ($MS_{A},MS_{e}$) are obtained by dividing the sums of squares by their corresponding degrees of freedom. Finally, the F statistic is constructed as $F=MS_{A}/MS_{e}$. By comparing the calculated F value with the critical value at a given significance level, it can be judged whether the factor has a significant impact on the test index: if the F value is greater than the critical value, the factor effect is significant, meaning that changes in factor levels indeed cause differences in the index values.

ANOVA can be used to draw two core conclusions: first, it indicates whether the impact of each factor on the test indicators is significant; second, it demonstrates the importance of each factor through comparing the mean squares or contribution rates of different factors. This method is widely used in experimental design and data analysis in various fields, providing a rigorous statistical basis for multi-factor comparison. Through ANOVA, real systematic effects can be identified from random fluctuations, thereby providing quantitative support for factor screening, parameter optimization, and mechanism interpretation.

According to the calculation requirements, in the analysis of variance for the three-factor and three-level orthogonal test adopted in this study, the degrees of freedom for both the sum of factor squares ($SS_{A}$) and the sum of error squares ($SS_{e}$) are 2. The significance level is set as α = 0.05, and the critical value of the F statistic obtained by examining the table is 19.00. Specifically, if the calculated F statistic value is greater than this critical value, it indicates that the corresponding factor has a significant impact on the test results. The detailed calculation process of ANOVA is shown in Equations (1)–(10).

In this study, the $L_{9}(3^{4})$ orthogonal table was used to arrange the experiments, and a total of *n* = 9 experiments were conducted. It should be noted that the observation result of the i-th test is y_i_ (i = 1, 2, …, 9). First, the following basic statistics were calculated.

Total Test Results (*T*): The sum of all observation values.

(1) $$T=\sum_{i=1}^{n}y_{i}$$

Total Mean (*ȳ*): The arithmetic mean of all observation values.

(2) $$\bar{y}=\frac{T}{n}$$

Correction Term (*CT*): A correction factor used to simplify the calculation of subsequent sums of squares.

(3) $$CT=\frac{T^{2}}{n}$$

The sum of squares of deviations reflects the degree of fluctuation or variation in the data. According to the additivity principle of ANOVA, the total variance can be decomposed into the variance caused by various factors and the variance caused by random errors.

Total Sum of Squares of Deviations ($SS_{T}$): This represents the fluctuation magnitude of all test data relative to the total mean, with its degree of freedom $df_{T}=n-1=8$.

(4) $$SS_{T}=\sum_{i=1}^{n}(y_{i}-\bar{y}{)}^{2}=\sum_{i=1}^{n}y_{i}^{2}-CT$$

Sum of Squares of Deviations for Each Factor (taking Factor A as an example)

For Factor A (with three levels), arranged in one column of the orthogonal table, the sum of squares of deviations $SS_{A}$ reflects the variation in the index caused by changes in the levels of Factor A.

Calculate the sum of indicators of Factor A at each same level. Let $K_{A1},\;K_{A2},\;K_{A3}$ represent the sums of test results corresponding to Level 1, Level 2, and Level 3 of Factor A, respectively. Due to the balance of the orthogonal table, each level appears the same number of times, denoted as r (r=n/3=3).

(5) $$SS_{A}=\frac{1}{r}\left(K_{A1}^{2}+K_{A2}^{2}+K_{A3}^{2}\right)-CT$$

The degree of freedom of Factor A is $df_{A}=3-1=2$. Similarly, the sums of squares of deviations for Factors B and C ($SS_{B}$ and $SS_{C}$) can be calculated, with their degrees of freedom both being 2.

If the level sums of the error column in the $L_{9}(3^{4})$ orthogonal table are $K_{e1},\;K_{e2},\;K_{e3}$, then the sum of squares of error deviations $SS_{e}$ is

(6) $$SS_{e}=\frac{1}{r}\left(K_{e1}^{2}+K_{e2}^{2}+K_{e3}^{2}\right)-CT$$

The degree of freedom of error is $df_{e}=3-1=2$. According to the additivity of sum of squares decomposition, $SS_{T}=SS_{A}+SS_{B}+SS_{C}+SS_{e}$ holds, which can be used to verify the accuracy of the calculation.

The magnitude of the sum of squares of deviations is affected by the degree of freedom. To eliminate this influence, the concept of the mean square is introduced. The mean square (*MS*) is defined as the ratio of the sum of squares of deviations to its corresponding degree of freedom, and it is an unbiased estimator of the population variance.

(7) $$MS_{A}=\frac{SS_{A}}{df_{A}},\;MS_{e}=\frac{SS_{e}}{df_{e}}$$

F Test and Significance Judgment

The F test determines whether a factor has a significant impact on the test results by comparing the mean square of the factor with the mean square of the error. Taking Factor A as an example, the *F* statistic is constructed as follows:

(8) $$F_{A}=\frac{MS_{A}}{MS_{e}}$$

In this study, the significance level α = 0.05, and the critical value F_0.05_(2, 2) = 19.00 was obtained by looking up the F distribution table. The judgment criteria are as follows:

If $F_{A}>F_{0.05}(2,\;2)$, then p<0.05, indicating that Factor A has a significant effect on the test index;

If $F_{A}\leq F_{0.05}(2,\;2)$, then p>0.05, indicating that the influence of Factor A is not significant.

Similarly, the F test can be performed for Factors B and C.

To further compare the relative importance of each factor and eliminate the interference of random errors on effect estimation, the sum of pure deviations of each factor and its contribution rate to the total variation were calculated.

The sum of pure deviations ($S{S_{A}}^{\prime }$) of Factor A is the residual value after eliminating the random error part from its sum of squares of deviations:

(9) $$S{S_{A}}^{\prime }=SS_{A}-df_{A}\times MS_{e}$$

The contribution rate ($\rho_{A}$) of Factor A is defined as the proportion of the sum of pure deviations to the total sum of squares of deviations:

(10) $$\rho_{A}=\frac{S{S_{A}}^{\prime }}{SS_{T}}\times 100\%=\frac{SS_{A}-df_{A}\cdot MS_{e}}{SS_{T}}\times 100\%$$

The contribution rate directly reflects the actual influence of the factor on the test results; the higher the contribution rate, the more important the influence of the factor on the index. The calculation method for the contribution rates for Factors B and C is the same as that for Factor A, and the corresponding observation values can be input during the calculation. The error contribution rate ($\rho_{e}$) can be calculated as $\rho_{e}=1-(\rho_{A}+\rho_{B}+\rho_{C})$, and its magnitude reflects the comprehensive influence of test accuracy and uncontrolled factors.

### 2.3. SHPB Test Principles

**Figure 1:**
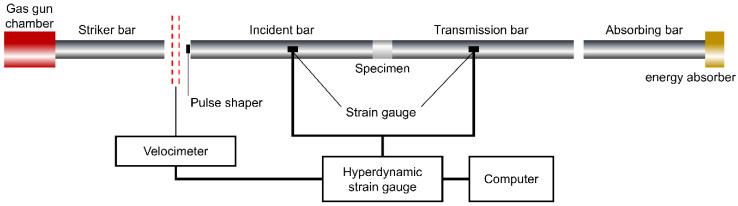
Schematic diagram of SHPB.

As illustrated in Figure 1, the split Hopkinson pressure bar (SHPB) test setup comprises an air gun chamber, striker bar, incident bar, transmission bar, absorbing bar, energy absorber, and data acquisition system. During the test, a single specimen is clamped between the incident and transmission bars. Driven by the air pressure in the air gun, the striker bar is propelled forward and impacts the incident bar, generating a pressure pulse in the incident bar; the pressure wave propagates forward in the incident bar. When propagating to the contact surface between the incident bar and the specimen, due to the inertial effect of the specimen and the bar material, the specimen is compressed, and, owing to the difference in wave impedance between the specimen and the incident bar, a portion of the pressure wave is reflected at the incident bar–specimen interface, generating a tensile wave that propagates in the reverse direction along the incident bar. The remaining portion of the pressure wave transmits through the specimen and propagates forward along the transmission bar via the specimen–transmission bar interface until it is absorbed by the energy absorber. In the process of longitudinal wave extension and bar and specimen propagation, strain gauges are affixed to the incident and transmission bars at equal distances from the specimen to record the stress waves in the bars [48]. By processing the incident, reflected, and transmitted waves collected by the strain gauges, the dynamic mechanical parameters of the specimen can be derived.

For the stress wave data acquired from the tests, the three-wave method [49] was employed to calculate the loading strain rate, dynamic compressive strength, stress–strain relationship, and energy absorption capacity of each of the specimens. The calculation equations are shown in Equations (11)–(15). It is worth noting that, in the SHPB test, the fragments produced after the specimen fails under an impact load will exhibit a splashing phenomenon. The kinetic energy of the fragments typically accounts for less than 5% [26,50,51] of the total energy absorbed by the specimen. The material absorption energy calculation equation is as follows:

(11) $$\left\{\begin{array}{c} \sigma_{s}\left(t\right)=\frac{EA_{0}}{{2A}_{s}}[\varepsilon_{i}\left(t\right)+\varepsilon_{r}\left(t\right)+\varepsilon_{t}\left(t\right)]\\ \varepsilon_{s}(t)=\frac{C_{0}}{l}\int_{0}^{t}[\varepsilon_{i}\left(t\right)-\varepsilon_{r}\left(t\right)-\varepsilon_{t}\left(t\right)]dt\\ {\dot{\varepsilon }}_{s}(t)=\frac{C_{0}}{l}\left[\varepsilon_{i}\left(t\right)-\varepsilon_{r}\left(t\right)-\varepsilon_{t}\left(t\right)\right] \end{array}\right.$$

(12) $$W_{i}=A_{0}EC_{0}\int_{0}^{t}\varepsilon_{i}^{2}\left(t\right)dt$$

(13) $$W_{r}=A_{0}EC_{0}\int_{0}^{t}\varepsilon_{r}^{2}\left(t\right)dt$$

(14) $$W_{t}=A_{0}EC_{0}\int_{0}^{t}\varepsilon_{t}^{2}\left(t\right)dt$$

(15) $$W_{a}=W_{i}-W_{r}-W_{t}$$

where *E* is the elastic modulus of the SHPB, *A*_0_ is the end area of the SHPB, *A_s_* is the end area of the specimen, *ε_i_* is the incident wave strain, *ε_r_* is the reflected wave strain, *ε_t_* is the transmitted wave strain, *C*_0_ is the wave propagation velocity in the SHPB, $C_{0}=\sqrt{\frac{E}{\rho }}$, *ρ* is the density of the SHPB, *l* is the specimen length, $\sigma_{s}(t)$ is the stress, $\varepsilon_{s}(t)$ is the strain, ${\dot{\varepsilon }}_{s}(t)$ is the strain rate, *W_i_* is the energy carried by the incident wave, *W_r_* is the energy carried by the reflected wave, *W_t_* is the energy carried by the transmitted wave, and *W_a_* is the energy absorbed by the specimen.

### 2.4. Test Setup

**Figure 2:**
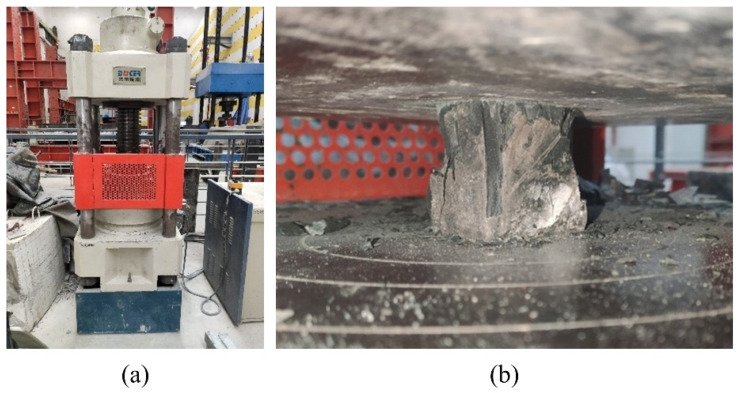
Quasi-static mechanics experimental setup. (**a**) MTS testing machine; (**b**) static uniaxial compressive test.

As shown in Figure 2, quasi-static compression tests were performed on a 600 kN MTS testing machine. For the static compression tests, specimens were installed following standard loading procedures, with a loading rate of 0.2 mm/min (corresponding to a strain rate of 6 × 10^−5^ s^−1^), which satisfied the quasi-static loading criteria [45]. The data acquisition frequency was 5 Hz.

**Figure 3:**
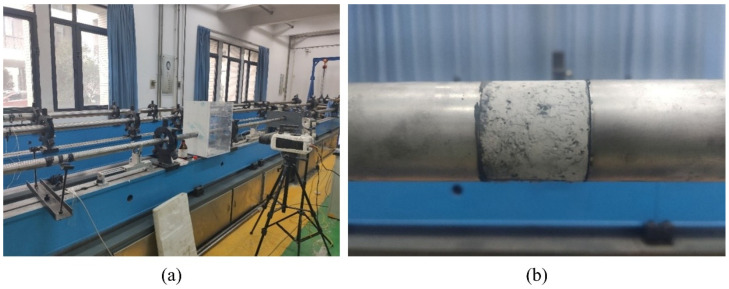
Dynamic mechanics experimental setup. (**a**) SHPB test system; (**b**) specimen setup.

As shown in Figure 3, dynamic impact tests were carried out by adjusting the air pressure of the air gun to propel the striker bar at different velocities toward the incident bar, thus resulting in different strain rates on the specimen. During the tests, three air pressure levels (0.3 MPa, 0.4 MPa, and 0.5 MPa) [48] were selected, corresponding to striker bar impact velocities of approximately 6.6 m/s, 7.4 m/s, and 8.0 m/s, respectively, with strain rates ranging from 70 to 180 s^−1^. To mitigate the dispersion effect induced by the rectangular wave propagating in the bars, a rubber sheet (12 mm in diameter and 4 mm in thickness) was affixed to the front end of the incident bar for stress wave shaping. During the tests, the two ends of each specimen were closely attached to the ends of the incident and transmission bars of the SHPB setup, respectively. Three specimens were tested for each group under each loading pressure condition. The average values were used for calculating the relevant parameters during the data processing phase. After loading, the specimen fragments were collected, sieved, and weighed to determine the particle size distributions of the fractured specimens.

## 3. Experimental Results

### 3.1. Quasi-Static Test Results

**Figure 4:**
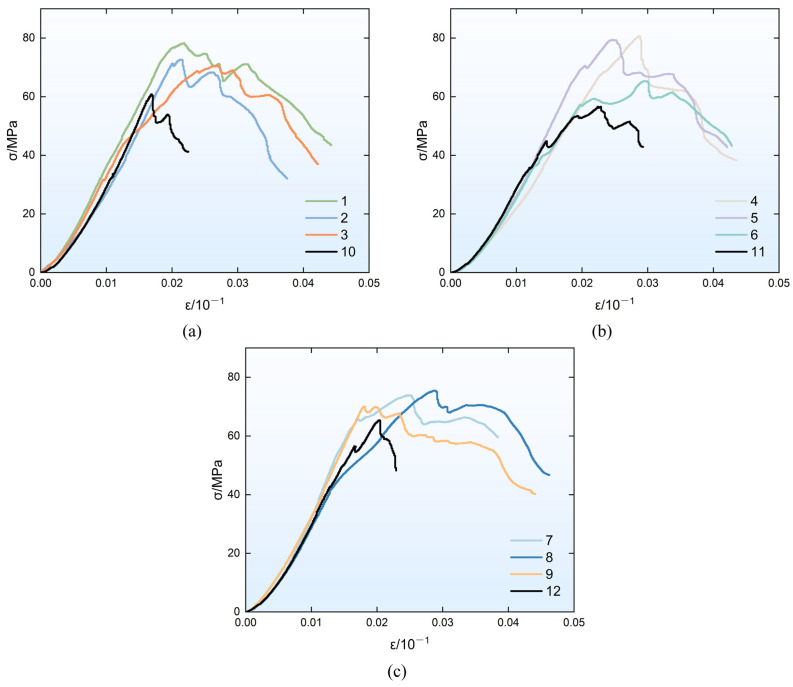
Stress–strain curves under quasi-static test. (**a**) Ash-slag ratio 7:3 (**b**) Ash-slag ratio 5:5 (**c**) Ash-slag ratio 4:6.

Figure 4 presents the quasi-static compressive stress–strain curves for each group of specimens. It can be observed from the figure that fiber addition significantly enhances the quasi-static compressive strength of the material. Compared with the plain geopolymer matrix, the quasi-static compressive strength of the composites with different fly ash/slag ratios is increased by an average of 21.43%, 32.82%, and 11.9%, respectively. Such strength improvements are closely associated with the ability of the fibers to restrict crack propagation and maintain the structural integrity of the material. Macroscopically, crack development in fiber-reinforced geopolymer composites proceeds more slowly under quasi-static loading, and the material can still retain a certain load-bearing capacity after reaching the peak stress, which is reflected by the presence of a post-peak plateau in the stress–strain curve.

It is worth noting that the quasi-static compressive strength of Group 11 specimens is slightly lower than that of Group 10; the reason may be as follows. Under a fixed alkali-equivalent ratio, the concentrations of soluble silicon, aluminum, and calcium ions in the system may reach a critical imbalance point when the fly ash/slag ratio is 5:5. In this case, OH^−^ ions may preferentially combine with Ca^2+^ ions to form Ca(OH)_2_ microregions or promote carbonate precipitation, thereby weakening the depolymerization capacity of the fly ash glass network. This leads to a decrease in the reaction efficiency of the geopolymer and the failure to form a sufficiently dense and uniform gel structure [52,53]. Meanwhile, when the fly-ash-to-slag ratio is 5:5, the slag may release a large amount of Ca^2+^ ions prematurely and at an excessively fast rate, which may inhibit the depolymerization of the Si-O-Al network in the fly ash and its subsequent condensation reaction. This competitive effect results in the insufficient formation of geopolymer gels (N-A-S-H) and may induce the generation of impurities, thereby reducing the densification degree and bonding strength of the material structure and further affecting the macroscopic compressive strength [54,55]. Existing studies also confirm that, when the fly ash/slag ratio reaches 5:5, its static compressive strength is lower than that under other ratios [52,53].

### 3.2. Damage Morphology

**Figure 5:**
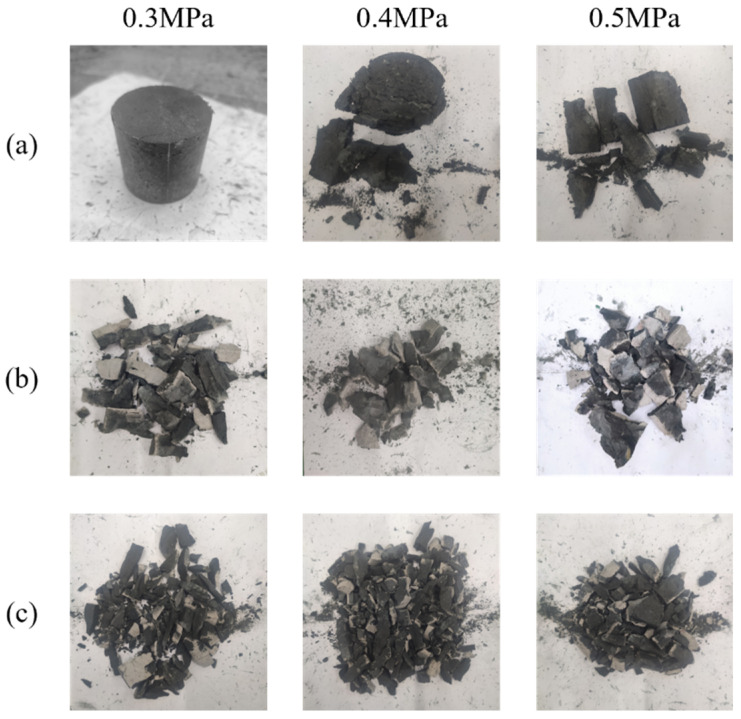
Fragments of specimens with different fragmentation degrees after dynamic impact: (**a**) relatively intact (Group 8); (**b**) moderately fractured (Group 3); (**c**) severely fractured (Group 10).

Figure 5 depicts the failure pattern characteristics of specimens from typical groups under dynamic compressive impact loading at different strain rates. It can be observed that, with increasing strain rates, the failure mode of the specimens generally evolves from longitudinal through-cracking to splitting into pieces and finally to complete fragmentation. Moreover, after failure, the specimen fragments peel off sequentially from the periphery toward the center, with the central region retaining relatively good integrity. This is because, under the impact load of the SHPB, the stress in the specimen is not one-dimensional. Due to inertia, the lateral deformation of the central region is constrained by the outer region, leading to a confining pressure effect that increases with the loading strain rate. Figure 5 presents the fragments of specimens with different fragmentation degrees (i.e., relatively intact, moderately fractured, and severely fractured). It can be found that the degree of breakage of the plain geopolymer matrix specimens is significantly greater than that of the fiber-reinforced geopolymer; for the same proportion of fiber-reinforced geopolymer, the degree of breakage gradually increases with an increase in loading pressure. Under the three levels of loading pressure, the specimens in Group 8 have the smallest degree of breakage.

**Figure 6:**
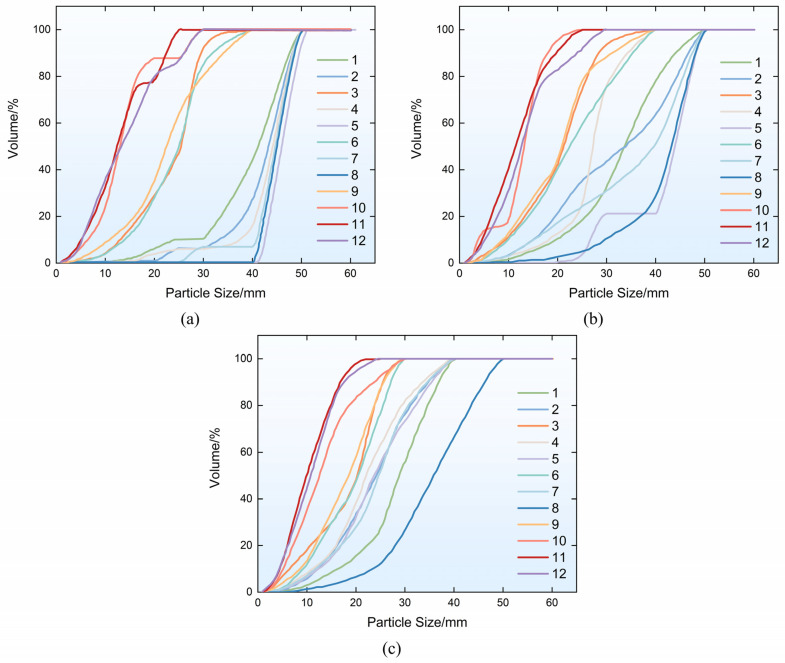
Particle size distribution curves of fragments for each group: (**a**) 0.3 MPa; (**b**) 0.4 MPa; (**c**) 0.5 MPa.

The specimen fragments were sieved using a set of standard sieves with pore diameters of 1 mm, 2.5 mm, 5 mm, 10 mm, 16 mm, 20 mm, 25 mm, 30 mm, and 40 mm, respectively. The particle size distribution curves of the sieved fragments are presented in Figure 6. An analysis of the figure indicates that the specimen fragments can primarily be classified into three distinct categories based on the particle size. At 0.3 MPa, specimens from Groups 1, 2, 4, 5, 7, and 8 were relatively intact; at 0.4 MPa, specimens from Groups 1, 2, 5, 7, and 8 were relatively intact; and, at 0.5 MPa, specimens from Group 8 were relatively intact, with over 50% of the fragments having a particle size greater than 40 mm. At 0.3 MPa pressure, Groups 3, 6, and 9 were relatively fragmented; at 0.4 MPa pressure, Groups 3, 4, 6, and 9 were relatively fragmented; and, at 0.5 MPa pressure, except for Group 8, all fiber-reinforced geopolymer specimens were relatively fragmented, with over 50% of the fragments having a particle size of less than 30 mm and with less than 30% having a particle size of less than 16 mm. Specimens from Groups 10, 11, and 12 (without fiber addition) exhibited severe fragmentation under impact loading at all three pressure levels, with over 50% of the fragments having a particle size of less than 16 mm.

The weighted average of the particle size content of the sieved fragments was defined as the average particle size ($d_{a}$) of the fragments in each group, and the calculation formula is as follows:

(16) $$d_{a}=\frac{\sum r_{i}d_{i}}{\sum r_{i}}$$

where $d_{i}$ denotes the particle size of each fraction, and $r_{i}$ represents the mass percentage of fragments within each particle size fraction.

**Figure 7:**
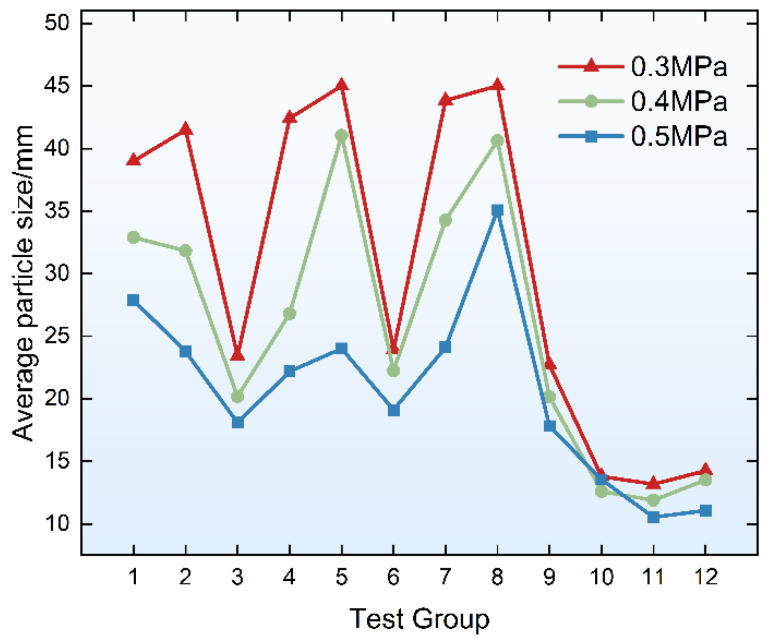
Average particle sizes of specimen fragments.

The average particle sizes of the specimen fragments for each group are shown in Figure 7. It can be seen from the table that the average particle size of the specimen fragments decreases with increasing loading air pressure. Additionally, the average particle sizes of fragments from Groups 10, 11, and 12 (without fiber addition) are significantly smaller than those of fiber-reinforced specimens with the corresponding matrix ratios. It can be observed from Figure 7 that the average particle size of each group of PVA-FRGC specimens is larger than that of the plain geopolymer matrix, indicating better integrity under dynamic compressive loading. This confirms that the addition of fibers effectively enhances the integrity of the material under dynamic compressive loading. As previously reported, PVA-FRGC exhibit obvious strain hardening and multi-crack characteristics under quasi-static loading, which is achieved through the bridging effect of fibers on the geopolymer matrix. Therefore, the improved integrity of the specimens under dynamic impact loading may also be attributed to the inhibitory effect of fibers on the propagation of material cracks, and this inhibitory effect is macroscopically reflected in the higher integrity of the material after loading. Meanwhile, it is noted that the average particle size in Groups 3, 6, and 9 is significantly smaller than those of other PVA-FRGC groups. This may be because these groups adopted 12 mm PVA fibers; during the debonding and sliding process, the excessive force between the long fibers and the matrix material exceeds the tensile strength of the fibers, leading to premature fiber fracture. As a result, the fibers fail to fully exert their inhibitory effects on crack propagation, allowing cracks to develop completely, which is manifested by the small average particle sizes of the specimens.

**Figure 8:**
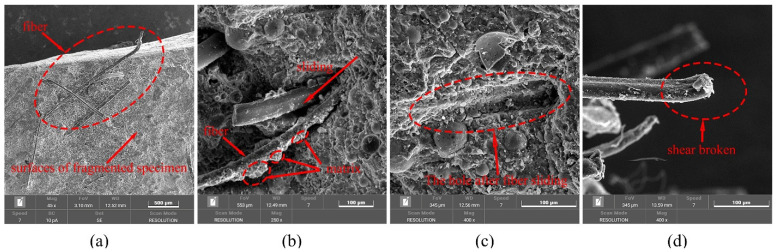
Fibers on the surfaces of specimen fragments: (**a**) surface of specimen; (**b**) sliding fibers; (**c**) hole after fiber sliding; (**d**) shear-broken fiber.

Figure 8a illustrates the exposed fibers on the fracture surfaces of fragmented PVA-FRGC specimens after dynamic compressive loading. As observed from the figure, during dynamic compression-induced fracture, the fibers were pulled out from the matrix, with an exposed length of approximately 1 mm. As shown in Figure 8b,c, the pulled-out fibers remained adhered to the surrounding matrix material, establishing interfacial interactions with the matrix throughout the pull-out process. Macroscopically, these fiber–matrix interactions contribute to the toughening effect of the composite. Additionally, Figure 8d reveals that the fractured fiber ends are clean and shear-dominated, indicating that the reinforcement mechanism of the fibers under dynamic compression is primarily governed by their pull-out behavior from the matrix. The intrinsic tensile strength of the fibers themselves exerts a negligible influence on the dynamic compressive response of the material. Therefore, it can be inferred that the reinforcing effect of fibers in the composite mainly depends on the interfacial mechanical properties between the fibers and the matrix, which are controlled by the fiber tensile strength, fiber–matrix debonding strength, and fiber–matrix physical adhesion strength. This phenomenon is similar to the failure mechanism observed under quasi-static loading conditions. Under quasi-static loading, fiber-reinforced geopolymer composites typically undergo a sequential failure process, including matrix cracking, fiber–matrix debonding, fiber pull-out, and eventual fiber fracture with crack propagation. The strain-hardening behavior exhibited by the material under quasi-static loading can be attributed to the gradual increase in interfacial sliding resistance during the fiber pull-out stage. In contrast, under dynamic loading, the processes of fiber–matrix debonding, fiber pull-out, and fiber fracture occur at an extremely high rate. Consequently, the fiber pull-out stage cannot provide sufficient interfacial sliding resistance to produce macroscopic strain-hardening characteristics. Nevertheless, the crack-restraining effect of fibers remains pronounced. Macroscopically, compared with unreinforced geopolymer matrix specimens, PVA-FRGC exhibit better structural integrity, higher strength, and an improved energy absorption capacity under dynamic impact loading, as will be discussed in detail in subsequent sections.

### 3.3. Analysis of Experimental Results Regarding Dynamic Compressive Strength

**Figure 9:**
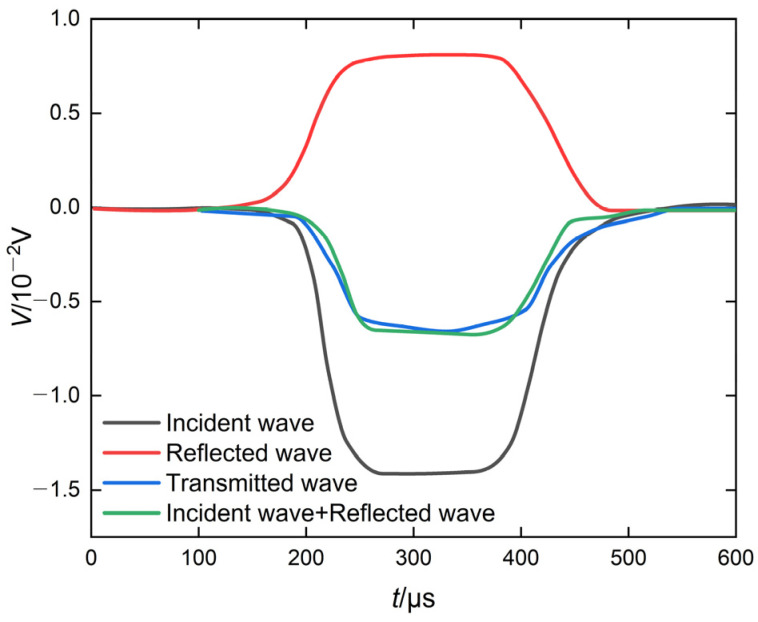
Original waveform after shaping.

The original waveform of PVA-FRGC under dynamic impact loading is presented in Figure 9, with Group 5 taken as an example. As observed from the figure, the waveform is well shaped, as described in Section 2.4, indicating the validity of the test results.

**Figure 10:**
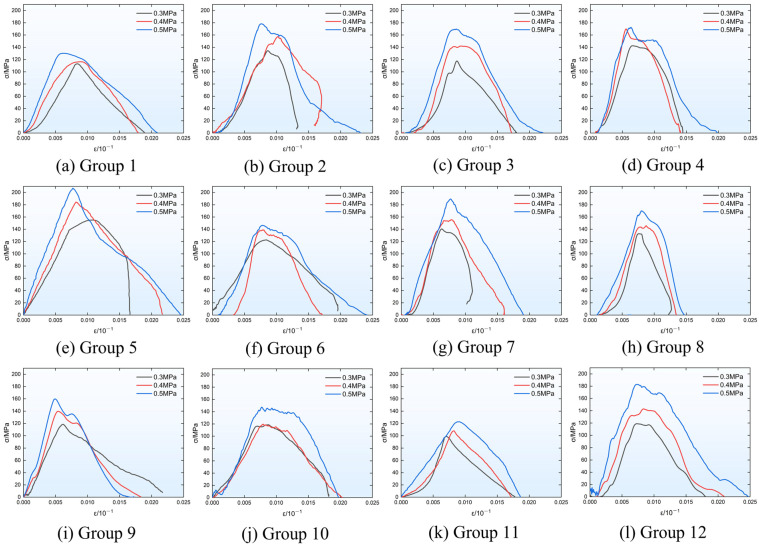
Stress–strain curves under dynamic test.

**Figure 11:**
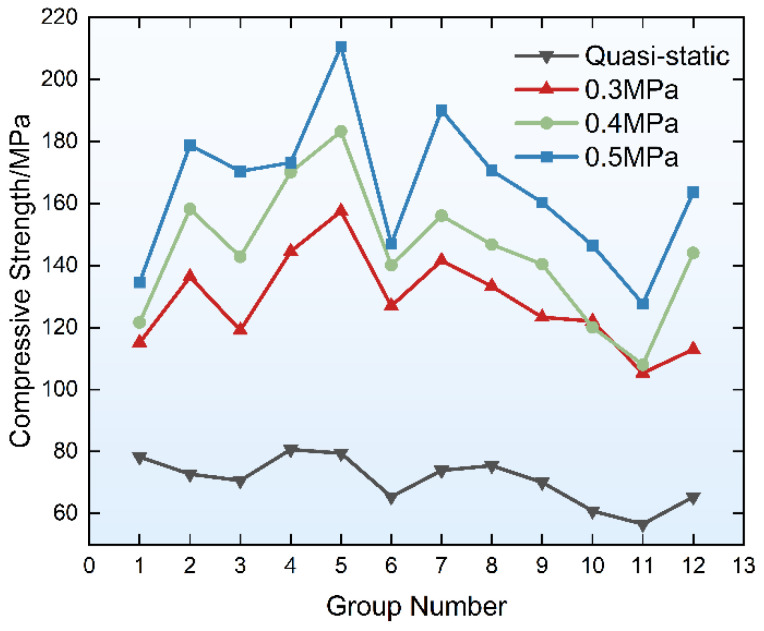
Compressive strength of different test specimens.

The specimens exhibited the same mechanical behaviors as those observed in the quasi-static tests, namely strain hardening and post-peak ductility [56,57,58]. Figure 10 presents the full-range stress–strain curves of PVA-FRGC specimens from each group under different impact pressure levels, while Table 2 lists the peak strength values of specimens from each group under the corresponding pressure levels. As observed from the curves, as the strain rate increases, the peak stress of specimens from each group increases gradually, and the elastic modulus also exhibits a similar increasing trend. At the three dynamic loading pressure levels, the maximum strength was recorded in Group 5, reaching 157.52 MPa, 183.26 MPa, and 210.68 MPa, respectively. The gradual strain softening in the post-peak stage indicates that the specimens possess high toughness, and the failure process is non-brittle. This further confirms that the specimens absorb energy primarily through this failure process. From the stress–strain curves of specimens from each group under an air pressure of 0.5 MPa, it can be observed that, in the post-peak softening region, the curves exhibit a softening plateau. Specifically, during this stage, the strain of the specimens continues to increase, while the post-peak strength degradation stagnates. This phenomenon indicates that the specimens possess post-peak workability not exhibited by ordinary concrete; they retain considerable strength even after failure or under large strains. Furthermore, this phenomenon is not obvious at low strain rates but becomes increasingly significant as the strain rate increases. Based on the peak strength of each group of specimens shown in Figure 11, compared with plain geopolymer matrix specimens (without fiber addition), PVA-FRGC exhibit a significant enhancement in dynamic compressive strength under impact loading, with average increases of 90.34%, 113.81%, and 144.41% under the three air pressure levels, respectively.

This study employed the same method as Yang X. [59] to perform an orthogonal experimental variance analysis on the test results of each group. The dynamic compressive strength of specimens from each group is listed in Table 2. An analysis of variance (ANOVA) was performed on the dynamic compressive strength test results of the specimens under three different loading pressures (0.3 MPa, 0.4 MPa, and 0.5 MPa). The analysis results are shown in Table 3, Table 4 and Table 5. In the tables, K_1_, K_2_, and K_3_ represent the sums of the test results corresponding to Levels 1, 2, and 3 of different factors, respectively; F represents the F statistic of the analysis of variance; and the contribution rate represents the degree of influence of each factor on the corresponding test results. The calculation methods for these parameters are described in Section 2.2, and the sequence is arranged according to their degree of influence. The optimal scheme of each factor is determined according to the level corresponding to the largest K value. It can be found that, under the three different loading levels, all three factors involved in this study (fly ash/slag ratio, fiber length, and fiber volume content) exert significant effects on the dynamic compressive strength of the specimens, with the corresponding F statistic values all greater than the critical F value. With an increase in loading pressure, the contribution rate of the fiber volume content to the dynamic compressive strength of the material gradually increases, while the contribution rate of the fly ash/slag ratio decreases progressively. The order presented in the table corresponds to the relative influences of the three factors from large to small under the corresponding loading level. It can be further observed that, when the loading pressure is 0.3 MPa, the relative contribution rates of the ash/slag ratio and fiber length to the dynamic compressive strength of the specimens are essentially the same, with that of the ash/slag ratio being slightly higher; the relative contribution rate of the fiber volume content is the lowest, which is consistent with the behavior of the material under quasi-static loading. Although fiber incorporation can restrain crack development, its contribution is limited at low strain rates. The dynamic compressive strength of the material still depends more on the matrix strength. However, due to the interaction between the fibers and the matrix, fiber addition may prevent the dynamic compressive strength of the matrix with an ash/slag ratio of 5:5 from decreasing (which would otherwise occur due to ion imbalance in the system). Meanwhile, it contributes to the matrix achieving higher dynamic compressive strength. When the dynamic impact load level increases, the relative contribution rate of the fiber volume content to the dynamic compressive strength of the material starts to increase, while that of the ash/slag ratio starts to decrease. With an increase in strain rate, during specimen failure, fibers begin to effectively debond and slide, thereby restraining crack development and enabling the specimens to develop greater strength. When the loading pressure reaches 0.5 MPa, the order of the relative contribution rates of each factor from largest to smallest is as follows: fiber volume content, fiber length, and ash/slag ratio. Under the three loading levels, when the fly-ash-to-slag ratio = 5:5, fiber length = 9 mm, and fiber volume content = 2% (i.e., the factor combination A2B2C3), the dynamic compressive strength of the specimens reaches the maximum value within the experimental range, achieving 157.52 MPa, 183.26 MPa, and 210.68 MPa under different loading levels, respectively. With an increase in dynamic loading, the dynamic compressive strength of the material depends more on the fiber volume content, followed by the fiber length; although the fly ash/slag ratio also shows a significant effect, its relative influence gradually decreases. It was found that, at loading pressures of 0.3 MPa, 0.4 MPa, and 0.5 MPa, the average strain rates of the specimens were 87.4 s^−1^, 117.56 s^−1^, and 167.94 s^−1^, respectively. The dynamic compressive strength of specimens from Group 5 was the highest among all groups under the three loading levels, reaching 157.52 MPa, 183.26 MPa, and 210.68 MPa, respectively. The factor combination for Group 5 specimens was A2B2C3 (fly-ash-to-slag ratio = 5:5, fiber length = 9 mm, fiber volume content = 2%). Furthermore, the local optimal mix ratio derived from the orthogonal analysis under all loading levels was consistent with this combination. The only difference was that the significance levels of the three factors varied with the loading level. At a loading pressure of 0.3 MPa, the fly-ash-to-slag ratio exhibited the highest significance level; at 0.4 MPa and 0.5 MPa, the fiber volume content had the highest significance level. The aforementioned strain rates were obtained by averaging the peak strain rates from the strain rate–time curves calculated using Formula (1). Based on the aforementioned analysis results, it can be concluded that, under different strain rate loading conditions, the local optimal mix ratio is a fly-ash-to-slag ratio of 5:5, a fiber length of 9 mm, and fiber volume content of 2%.

Based on the experimental results of this study, the specimens in Group 5 yielded the local optimal dynamic mechanical properties among the selected parameter combinations, and this outcome is consistent with the predictions from the analysis of variance (ANOVA). The specific mix proportion of Group 5 is as follows: a fly ash/slag ratio of 5:5, a fiber length of 9 mm, and fiber volume content of 2.0%. Although the plain geopolymer matrix exhibits lower strength at a 5:5 fly ash/slag ratio, the incorporation of fibers effectively mitigates this limitation. Under loading, the fibers activate their bridging effect at an early stage of crack propagation, which efficiently restricts crack opening and the propagation velocity, thereby conferring superior macroscopic strength and crack resistance to the composite. Regarding the fiber length, excessively short fibers tend to pull out or fracture prematurely during the sliding and frictional processes associated with crack inhibition, resulting in an insufficient bridging effect. Conversely, overly long fibers experience excessive interfacial interaction forces with the matrix during sliding, which exceed the tensile strength of individual fibers. This prevents effective fiber debonding and sliding, leading instead to premature fiber fracture. Therefore, fibers with a moderate length are the most effective at inhibiting crack propagation under dynamic impact loading and at maximizing the strength of the specimens. In this study, the optimal dynamic mechanical properties were achieved with a fiber length of 9 mm. The fiber volume content is the most prominent factor influencing the dynamic compressive mechanical properties of PVA-FRGC. Higher fiber volume content provides more fibers to exert the bridging effect during material failure, thereby enhancing the dynamic compressive strength. However, excessively high fiber volume content can lead to fiber agglomeration due to inadequate dispersion during the mixing process, which compromises the integrity of the matrix. The maximum fiber volume content selected in this study was 2.0%, which still exerted a positive effect on the dynamic compressive strength of the material. This suggests that the critical fiber volume content (beyond which agglomeration occurs) may be higher than the upper limit of the parameter range investigated in this study.

**Figure 12:**
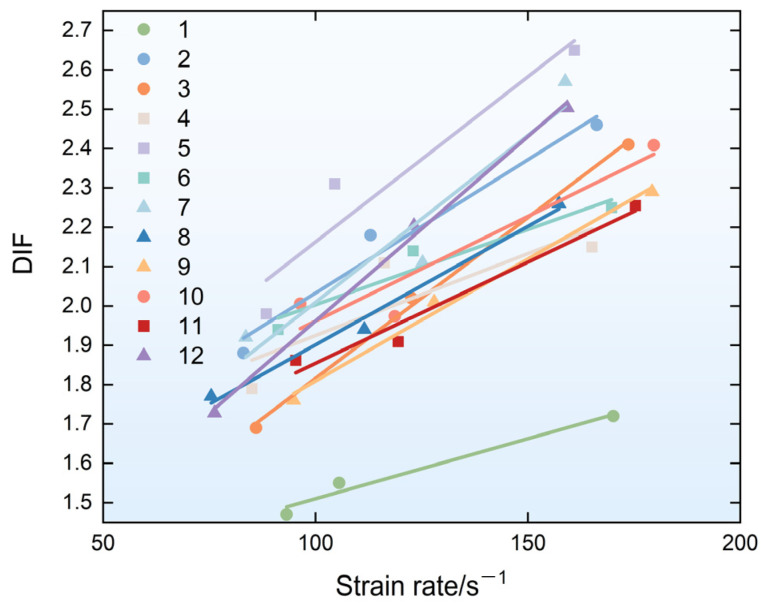
Dynamic increase factors.

The quasi-static compressive strength of the specimens after 28 days is listed in Table 2. The dynamic compressive strength is the stress value corresponding to the highest point of the stress–strain curve. The dynamic increase factor (DIF) is the ratio of the dynamic compressive strength to the quasi-static compressive strength, which can reflect the increase in the compressive strength of the specimens under impact loads. The equation is shown in Equation (17). The DIFs of each mix proportion are shown in Figure 12. It can be found that all groups exhibited significant strain rate sensitivity, with the addition of fibers noticeably improving the dynamic compressive strength of the materials. All specimens exhibited greater compressive strength under dynamic impact loading than quasi-static loading, and the increase in the DIF value for most groups increased with increasing strain rates.

(17) $$DIF=\frac{f_{d}}{f_{s}}$$

where *f_d_* is the dynamic compressive strength, and *f_s_* is the quasi-static compressive strength.

In some cases, the dynamic compressive strength of PVA-FRGC is even lower than that of the matrix without fibers. The reason is that the fiber itself does not have the ability to withstand compression, and the reinforcing effect on the material is mainly reflected in the case of limiting the development of cracks and tensile loads [60]. Moreover, the addition of fibers renders the matrix defective under compressive loads, so it affects the strength of the material, but the inhibition effect of the fiber on crack development in the process of material cracking is still obvious.

### 3.4. Analysis of Experimental Results Regarding Energy Absorption Capacity

**Figure 13:**
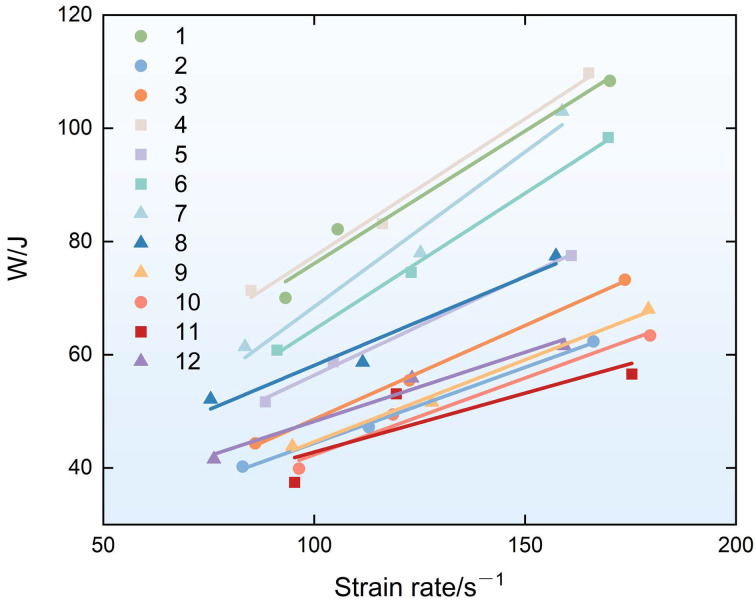
Energy absorption–strain rate curve.

An analysis of variance (ANOVA) was performed on the energy absorption capacity of the specimens under different strain rate loading levels. The analysis showed that each factor was significant under the loading pressures of 0.3 MPa, 0.4 MPa, and 0.5 MPa, and the significance ranking of factors followed the order of B > A > C. The relative contribution rate of the fiber length to energy absorption is much higher than those of the other two factors. As mentioned above, the longer the fiber length, the more likely it is that the bridging stress will exceed the tensile strength of the fiber during crack propagation, resulting in tensile failure before the fiber is completely pulled out. From the perspective of energy absorption, complete debonding and sliding processes are more conducive to energy absorption. Although the ash/slag ratio and fiber volume content also have significant effects on the energy absorption capacity of the material, their significance is much lower than that of the fiber length. Specifically, with an increase in the load level, the relative contribution rate of the fiber length increased from 67.34% to 73.22%, while that of the ash/slag ratio decreased from 16.2% to 12.37% and that of the fiber volume content decreased from 14.6% to 11.94%. As illustrated in Figure 13, compared with plain geopolymer matrix specimens (without fiber addition), the energy absorption capacity of PVA-FRGC under dynamic impact loading was not significantly improved. This may be attributed to the fact that plain geopolymer matrix specimens underwent more severe fragmentation under various strain rate conditions. Unlike PVA-FRGC, they lacked fiber confinement, leading to the formation of more cracks and consequently higher energy absorption. At the same impact load level, fibers did not significantly improve the energy absorption capacity of the specimens [28]. However, for specimens with similar energy absorption levels, PVA-FRGC exhibited significantly less fragmentation than plain geopolymer matrix specimens, which confirms the reinforcing effect of fibers. Under the same loading level, the specimens of Group 5 showed better integrity due to the addition of fibers, with average particle sizes that were 241.43%, 245.04%, and 127.80% higher than in the specimens of Group 11 without fibers, respectively. Among all groups, the average particle sizes of the specimen fragments in Group 5 showed the greatest increase compared with the plain geopolymer matrix specimens. As mentioned earlier, the addition of fibers may alter the ion imbalance in the matrix at an ash-to-slag ratio of 5:5, thereby forming better interfacial properties with the fibers. The 9 mm PVA fiber can achieve the maximum interfacial energy dissipation efficiency, balance the effective insertion and withdrawal of the fibers as well as their dispersion uniformity, and ensure the effective bridging and energy absorption of the fibers at cracks. Fiber volume content of 2.0% ensures that the fibers form a continuous energy dissipation network in the matrix, which maximally suppresses impact-induced meso-fragmentation and thus ensures a larger average particle size after dynamic impact.

In summary, PVA-FRGC exhibit superior dynamic impact resistance when the fly-ash-to-slag ratio is 5:5 and the PVA fiber length is 9 mm. Specifically, when the fiber volume content is 2.0%, the dynamic compressive strength of the specimens under different strain rate conditions increased by 49.53%, 69.64%, and 65.07%, respectively, compared to the plain geopolymer matrix specimens. The DIF values of the specimens reached 1.98, 2.31, and 2.65, respectively, under different load levels. More notably, the addition of fibers not only improved the dynamic compressive strength and energy absorption capacity of the material but also significantly improved its fracture performance—that is, the integrity of the material under dynamic impact loading was significantly improved. Taking Group 5 as an example, the average particle sizes were 241.43%, 245.04%, and 127.80% higher than in Group 11 with the same matrix ratio at various loading levels.

## 4. Discussion

The experimental results of this study deepen and expand the current understanding of the dynamic mechanical properties of PVA-FRGC. In contrast to existing research, alkali-activated geopolymers were adopted as the primary matrix material herein, rather than being incorporated as an admixture into ordinary concrete [31]. Moreover, orthogonal experiments were designed to investigate the coupled effects of multiple factors on the dynamic compressive mechanical properties of the material, as opposed to analyzing the influences of individual factors in isolation [32]. Existing research shows that the addition of PVA fibers effectively improves the dynamic mechanical properties of geopolymers [61,62]. By observing the failure morphologies of the specimens, it was found that cracks propagated along the generatrices during failure, eventually forming a core-like failure structure. This phenomenon occured because the loading rate in the experiment was relatively high, and the instantaneous failure of the specimen induced axial confining pressure. This loading condition was consistent with the dynamic impact loads experienced by materials in engineering practice, thus providing a reasonable explanation for the observed failure characteristics. Regarding the dynamic compressive strength, the three factors investigated in the test all exerted significant effects on the dynamic compressive strength of the material. When the loading level was low, the contribution rates of the fly ash/slag ratio and fiber length to the dynamic compressive strength of the specimens were basically the same, with that of the fly ash/slag ratio being slightly higher, while the contribution rate of the fiber volume content was the lowest. However, as the loading level increased, the contribution rate of the fiber volume content increased gradually, whereas that of the fly ash/slag ratio decreased. Specifically, when the loading pressure was 0.3 MPa, the relative contribution rate of the ash/slag ratio was 37.75%, that of the fiber length was 37.19%, and that of the fiber volume content was 20.68%. When the loading pressure was 0.5 MPa, the relative contribution rate of the fiber volume content to the dynamic compressive strength of the specimen reached 58.15%, the relative contribution rate of the fiber length was 30.11%, and the relative contribution rate of the fly ash/slag ratio was only 9.7%. When the material was subjected to dynamic compressive loading, the addition of fibers contributed significantly to inhibiting crack propagation: the fiber length determines the magnitude of the interaction force between a single fiber and the matrix during crack development, while the fiber volume content affects the macroscopic crack inhibition effect. In contrast, the fly ash/slag ratio simultaneously influences the strength of the matrix material and the interaction force between the matrix and fibers. This verifies the enhancing effect of fiber addition on the dynamic mechanical properties of the material. The dynamic compressive strength of the specimens showed a trend of first increasing and then decreasing with the increase in the slag content in the fly ash/slag mixture and the fiber length. It reached the maximum when the fly-ash-to-slag ratio was 5:5 and the fiber length was 9 mm, while it increased gradually with increasing fiber volume content, reaching the maximum at the fiber volume content of 2%. Observing the microstructures of the specimens served to further verify the role of fibers in improving the dynamic mechanical properties of the material. This material exhibited significant strain rate sensitivity [30]. Under the same load level, the energy absorption level of the material did not change significantly, but, due to the addition of fibers, its fragmentation degree was significantly reduced. Compared with the matrix material without added fibers, the average particle size of PVA-FRGC fragments increased by 135% after failure, which once again confirms the reinforcing effects of fibers on materials. The relationship between the energy absorption of PVA-FRGC and the degree of fragmentation found in this study can further deepen the understanding of the mechanical properties of this material under dynamic impact loading. In engineering applications, PVA-FRGC can maintain better structural integrity, thereby improving the stability of engineering structures under dynamic loads and enhancing engineering safety.

In this study, there were still some aspects that need to be further examined. For example, fiber volume content of 2% served as the boundary value for the parameters selected in this study. The dynamic compressive mechanical properties of the material may be improved with an increase in fiber volume content, which will be investigated in subsequent experimental and numerical simulation studies. The reinforcement mechanism of the interaction between PVA fibers and geopolymers with an ash/slag ratio of 5:5 also needs to be further studied. A numerical model of materials with different ratios needs to be established to study the dynamic mechanical properties of more mix ratios.

## 5. Conclusions

In this study, a three-factor and three-level orthogonal test was adopted to systematically investigate the effects of the fly-ash-to-slag ratio, fiber length, and fiber volume fraction on the dynamic compressive strength, energy absorption capacity, and fragmentation degree of PVA fiber-reinforced geopolymer composites (PVA-FRGC). The core conclusions are summarized as follows.

(1) Dynamic compressive strength and optimal mix proportion: The matrix precursor proportion (fly ash/slag ratio), fiber length, and fiber volume fraction all have significant effects on the dynamic compressive strength of PVA-FRGC (F statistic > critical F value at α = 0.05). With an increase in the loading strain rate, the contribution rate of the fly ash/slag ratio to the dynamic compressive strength decreases from 37.75% to 9.7%, while that of the fiber volume fraction increases from 20.68% to 58.15%. The local optimal mix proportion is determined as a fly ash/slag ratio of 5:5, fiber length of 9 mm, and fiber volume content of 2.0%; under the three different impact load levels, the dynamic compressive strength of specimens with these proportions is 157.52 MPa, 183.26 MPa, and 210.68 MPa, respectively. The optimal performance is mainly attributed to the better bridging effect between the fiber length and volume content and the matrix during the crack propagation process. At the same time, the material exhibits obvious strain rate sensitivity under all dynamic loading levels.
(2) Energy absorption capacity and fragmentation degree: The fly-ash-to-slag ratio, fiber length, and fiber volume content all significantly affect the energy absorption capacity of PVA-FRGC under dynamic impact loading, among which the fiber length exerts the most prominent influence. Notably, fiber incorporation does not significantly improve the energy absorption capacity of the material at the same strain rate level. However, for specimens with comparable energy absorption capacities, fiber reinforcement significantly reduces the fragmentation degree of the composite compared with plain geopolymer matrices: the average fragment particle size increases by 241.43%, 245.04%, and 127.80% under different loading levels, which confirms the effective crack inhibition effect of fibers.
(3) Failure mode and microscopic mechanism: Under dynamic compressive impact loads, the cracks of PVA-FRGC propagate along the generatrices of cylindrical specimens and peel off sequentially from the surface to the center, eventually forming fragments with a core-like structure. The addition of fibers significantly improves the integrity of the material after failure. Scanning electron microscopy (SEM) observations show that the material exhibits fiber debonding, sliding, and fracture mechanisms under dynamic loading, which are similar to those under static loading conditions, further verifying the stable reinforcing mechanism of PVA fibers in the geopolymer matrix.

## Figures and Tables

**Table 1:** Orthogonal mix proportions of samples.

Test Group	Fly-Ash-to-Slag Ratio	Fiber Length/mm	Fiber Volume Content/%
1	7:3	6	1.0
2	7:3	9	1.4
3	7:3	12	2.0
4	5:5	6	1.4
5	5:5	9	2.0
6	5:5	12	1.0
7	4:6	6	2.0
8	4:6	9	1.0
9	4:6	12	1.4
10	7:3	/	/
11	5:5	/	/
12	4:6	/	/

**Table 2:** Results of orthogonal test.

Test Group	Ash/Slag Ratio	Fiber Length/mm	Fiber Volume Content/%	Quasi-Static Compressive Strength/MPa	Dynamic Compressive Strength/MPa			Absorbed Energy/J		
					0.3 MPa	0.4 MPa	0.5 MPa	0.3 MPa	0.4 MPa	0.5 MPa
1	1	1	1	78.23	115.14	121.65	134.57	70.04	82.15	108.37
2	1	2	2	72.70	136.37	158.22	178.84	40.25	47.21	62.34
3	1	3	3	70.64	119.06	142.85	170.41	44.34	55.47	73.25
4	2	1	2	80.64	144.53	170.13	173.20	71.38	83.16	109.80
5	2	2	3	79.46	157.52	183.26	210.68	51.68	58.76	77.53
6	2	3	1	65.39	126.90	140.12	146.98	60.81	74.55	98.38
7	3	1	3	73.93	141.59	156.05	190.10	61.39	77.97	102.98
8	3	2	1	75.44	133.26	146.72	170.65	52.13	58.65	77.43
9	3	3	2	70.04	123.31	140.44	160.33	43.80	51.59	68.01
10	1	/	/	60.82	122.00	120.04	146.49	39.89	49.42	63.38
11	2	/	/	56.59	105.34	108.03	127.63	37.47	53.1	56.6
12	3	/	/	65.36	112.93	144.04	163.64	41.56	55.88	61.58

**Table 3:** Sensitivity analysis under 0.3 MPa.

Parameter	Compressive Strength (MPa)			Absorbed Energy (J)		
	Ash/Slag Ratio	Fiber Length	Fiber Volume Content	Ash/Slag Ratio	Fiber Length	Fiber Volume Content
K1	370.56	401.25	375.3	154.62	202.8	182.97
K2	428.94	427.14	404.22	183.87	144.06	155.43
K3	398.16	369.27	418.17	157.32	148.95	157.41
F	35.56	35.05	19.93	35.52	144.54	32.13
Contribution	37.75%	37.19%	20.68%	16.20%	67.34%	14.60%
Sequence	A > B > C			B > A > C		
Optimal Scheme	A2	B2	C3	A2	B1	C1

**Table 4:** Sensitivity analysis under 0.4 MPa.

Parameter	Compressive Strength (MPa)			Absorbed Energy (J)		
	Ash/Slag Ratio	Fiber Length	Fiber Volume Content	Ash/Slag Ratio	Fiber Length	Fiber Volume Content
K1	422.73	447.84	408.48	184.83	243.27	215.37
K2	493.5	488.19	468.78	216.48	164.61	181.95
K3	443.22	423.42	482.16	188.22	181.62	192.18
F	25.20	20.33	29.25	22.32	126.70	21.65
Contribution	31.91%	25.51%	37.28%	12.42%	73.22%	12.02%
Sequence	C > A > B			B > A > C		
Optimal Scheme	A2	B2	C3	A2	B1	C1

**Table 5:** Sensitivity analysis under 0.5 MPa.

Parameter	Compressive Strength (MPa)			Absorbed Energy (J)		
	Ash/Slag Ratio	Fiber Length	Fiber Volume Content	Ash/Slag Ratio	Fiber Length	Fiber Volume Content
K1	483.81	497.88	452.19	243.96	321.15	284.19
K2	530.85	560.16	512.37	285.72	217.29	240.15
K3	521.07	477.72	571.17	248.4	239.64	253.77
F	19.96	59.86	114.69	21.12	120.05	20.42
Contribution	9.70%	30.11%	58.15%	12.37%	73.22%	11.94%
Sequence	C > B > A			B > A > C		
Optimal Scheme	A2	B2	C3	A2	B1	C1

## Data Availability

The original contributions presented in this study are included in the article. Further inquiries can be directed to the corresponding author.
